# Mycelium: A Nutrient-Dense Food To Help Address World Hunger, Promote Health, and Support a Regenerative Food System

**DOI:** 10.1021/acs.jafc.3c03307

**Published:** 2023-12-06

**Authors:** Roberta R. Holt, John P. Munafo, Julie Salmen, Carl L. Keen, Behroze S. Mistry, Justin M. Whiteley, Harold H. Schmitz

**Affiliations:** † Department of Nutrition, University of California, Davis, Davis, California 95616, United States; ‡ Department of Food Science, University of Tennessee, Knoxville, Tennessee 37996, United States; § Nutritious Ideas, LLC, Saint John, Indiana 46373, United States; ∥ Meati Foods, 6880 Winchester Cir Unit D, Boulder, Colorado 80301, United States; ⊥ March Capital US, LLC, Davis, California 95616, United States; # T.O.P., LLC, Davis, California 95616, United States; ∇ Graduate School of Management, University of California, Davis, Davis, California 95616, United States

**Keywords:** mycelium, mycofoods, hunger, sustainability, protein, micronutrients

## Abstract

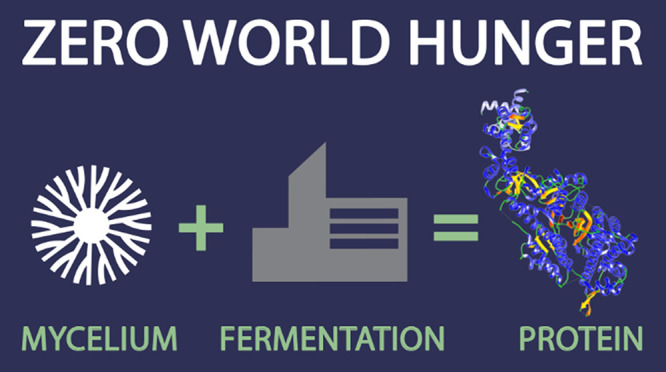

There is a need for transformational innovation within the existing food system to achieve United Nations Sustainable Development Goal 2 of ending hunger within a sustainable agricultural system by 2030. Mycelium, the vegetative growth form of filamentous fungi, may represent a convergence of several features crucial for the development of food products that are nutritious, desirable, scalable, affordable, and environmentally sustainable. Mycelium has gained interest as technology advances demonstrate its ability to provide scalable biomass for food production delivering good flavor and quality protein, fiber, and essential micronutrients urgently needed to improve public health. We review the potential of mycelium as an environmentally sustainable food to address malnutrition and undernutrition, driven by food insecurity and caloric dense diets with less than optimal macro- and micronutrient density.

## Introduction

The United Nations Sustainable Development Goal 2 (UNSDG2) requires a multidisciplinary approach to achieve its aim of ending hunger while providing food and nutrition security within a sustainable agricultural system by 2030.^1^ Achieving this goal requires transformational innovations that can be rapidly scaled given that as of 2020 approximately 720–811 million people suffer from hunger, with another 2.4 billion being moderately or severely food insecure.^2^ Malnutrition increasingly coexists as both undernutrition driven by food insecurity and obesity driven by nutrition insecurity in many regions of the world.^3,4^ This conundrum emphasizes the urgent need for the development of affordable foods desirable for global consumers and dense in bioavailable nutrients that are required for improving public health. In addition to the development of affordable foods providing better nutrition, an associated challenge will be manufacturing these foods within a food system that is environmentally beneficial and enables resilient agriculture practices.^5^

New developments with plant-based proteins have created opportunities to improve public health and environmental sustainability while reducing dependence on animal-based food products. Increasingly, dietary recommendations include a greater intake of plant-based foods to reduce noncommunicable chronic disease burden in both the developing and developed world.^6,7^ In addition to opportunities for improving public health, plant-based diets can lower the impact of food systems on the environment by reducing water use and the production of greenhouse gases.^8^ However, potential concerns of plant-based diets include the bioaccessibility of essential nutrients not produced endogenously or in sufficient quantity to support health that then must be obtained from the diet and “limiting” amino acids that are not present in sufficient quantity to stimulate protein synthesis. Representative of these concerns are populations with limited dietary protein diversity and a high incidence of anemia, stunting, and other health conditions associated with micronutrient deficiencies. Finally, any changes to an existing food system should be adaptable to the local environment and economic conditions and be sensitive to cultural practices.^9^

In addition to diets containing more plant-based foods, mycelium produced from filamentous fungi offers opportunities to develop food products that have desirable flavor and texture characteristics that are high in protein quality while providing fiber and essential micronutrients. Historically, not new to the food supply, fungal mycelium has gained interest, as technological advances have aided its formation into a protein biomass for food production. Some of these products are referred to as mycoprotein.^10,11^ Here, we review the potential of mycelium as a sustainable category of food well positioned to reduce malnutrition and enable the goal of zero hunger. We will first provide a basic understanding of mycelium while outlining the historical perspective of mycelium as a food. This historical perspective is followed by a discussion regarding the nutritional composition of mycelium, its potential benefits for public health, and future research needs in this context. Finally, we discuss the potential of mycelium-based foods, or mycofoods, as an affordable, scalable, and environmentally sustainable new source of high-quality protein for global consumers.

## Mycelium Basics

Fungi are one of the largest groups of eukaryotic organisms on the planet. They play many ecological roles in the environment including nutrient and carbon cycling and have been documented to be intimately interconnected with other organisms existing in mutualistic, pathogenic, and saprotrophic lifestyles.^12^ The fungal kingdom has enormous diversity, and recent estimates have indicated that the species count may range from two to 11 million species, with about 155,598 fungal species formally described to date.^13−15^ Analysis of metabarcoding data has suggested an even larger species number as high as 1.7–13.2 million species.^16^ Thus, fungi are considered one of the largest and least explored biodiverse resources on the planet.

Accordingly, there has been historical debate, more so than any other group of eukaryotic organisms, as to which groups to include or exclude within a taxonomical group. With advances in technology and expansion in knowledge over the years, most notably in genomics, there has been a shift in the phylogenic classification methodologies employed, moving from taxonomy based on mostly shared key morphological, ecological, and physiological characteristics, to more reliance on the similarity of relevant DNA sequences.^17^ In addition to a large diversity of species identified via DNA sequencing, fungi also have a diversity of morphological growth forms that are still in use for taxonomical classification. Prior to DNA sequencing, the historical reliance mostly on morphological characteristics added to the complexity of classification.^12^ For example, one characteristic that has aided in species identification is the size of reproductive structures. If the reproductive structure is visible to the naked eye (i.e., mushroom-forming fungi), the fungus is referred to as a macrofungus. If the reproductive structure is not visible to the naked eye (i.e., yeast), the fungus is referred to as a microfungus. Although considered as artifical taxonomic characters, the terminology is still useful for identification purposes and communication to the public. Additionally, teleomorph refers to fungi in the sexual state and anamorph refers to those in an asexual state. However, some fungi species have only been identified as anamorphs, with these species historically referred to as fungi imperfecti, demonstrating the complexity of a classification system based predominantly on morphological characteristics. With the implementation of modern molecular systematic methodologies, the taxonomy of fungi lacking distinct reproductive morphological structures has been better resolved.

Most commonly, fungi grow vegetatively in the form of elongated cells or hyphae that are often branching and tubelike in appearance. A network of hyphal cells growing together are referred to as mycelium. Within the different taxonomical groups of fungi, hyphae tend to be generally uniform, with some exceptions, for example, the presence or absence of cross-walls within hyphal cells, referred to as septa. In addition, not all fungi grow as hyphae but some grow as discrete single yeast cells. Some species are dimorphic and can switch between hyphal and yeastlike growth stages, with intermediate stages referred to as pseudohyphae. However, the term mycelium is not limited to fungi, but also occurs in other non-fungi organisms including those in Chromista.^12^ For the purpose of this review, we focus on fungal mycelium.

The kingdom fungi have undergone many phylogenetic revisions in the past century,^12^ with advances in high-throughput sequencing generating large amounts of meaningful DNA sequencing. Recent phylogenetic analysis has proposed 18 phyla, nested in 9 subkingdoms of fungi (Table 1).^17^ Within the fungi kingdom, the two main phyla most commonly used in food production are Basidiomycota and Ascomycota; however, some members of the lesser-known Mucoromycota such as *Mucor* species and *Rhizopus* species are also used in the production of fermented food products.

**Table 1:** 9 Subkingdoms and 18 Phyla of Fungi Proposed in 2018 by Tedersoo et al.^17^

subkingdom	phylum
holomycota	rozellomycota
aphelidiomyceta	aphelidiomycota
blastocladiomyceta	blastocladiomycota
chytridiomyceta	chytridiomycota
	monoblepharomycota
	neocallimastigomycota
olpidiomyceta	olpidiomycota
basidiobolomyceta	basidiobolomycota
zoopagomyceta	entomophthoromycota
	kickxellomycota
	zoopagomycota
mucoromyceta	mucoromycota
	mortierellomycota
	calcarisporiellomycota
	glomeromycota
dikarya	entorrhizomycota
	basidiomycota
	ascomycota

## Historical Perspective of Mycelium

Fungi have been identified in the fossil record that spans many different time periods in earth’s geological history. There are many reports of fungi in fossil records. Recently, fossilized fungal mycelium was discovered in sedimentary rocks in the Doushantuo Formation in Guizhou Province of China, from the Ediacaran period which is estimated to be from approximately 635 to 541 million years ago.^18^ In another study, fossilized structures were described that were morphologically consistent with that of fungi, preserved in the shale of the Grassy Bay Formation in Arctic Canada. These specimens were estimated to be possibly over 1 billion years old.^19^ In addition to the various ecological roles that fungi play, such as nutrient and carbon cycling, fungi provide a source of food for a diversity of organisms. There are many examples of fungi as a food source for organisms including plants, microbes such as bacteria, fungi, and ciliates, and animals including mollusks, insects, birds, and mammals. Fungi, such as truffles and mushrooms, may have once played a greater role as food for vertebrates; however, it has been hypothesized that as the chemical diversity of toxins in some fungal species increased, some mammals may have diversified their food sources to reduce fungal intake.^20^

Fungi play a major role in traditional food culture and society, with a long-documented history of intake of a variety of fungi by humans. As microfungi produce a suite of functional metabolites, fermented foods are a primary source of intake. Fermenting microfungi such as *Aspergillus oryzae* and yeasts such as *Zygosaccharomyces sp., Brettanomyces sp., and Saccharomyces sp.* often produce metabolites that preserve food that can increase food safety, such as ethanol, 2,3 -butanediol, and 2-phenylethanol. They can also produce various organic acids, such acetic acid, propanoic acid, and butanoic acid. These metabolites not only inhibit spoilage and pathogenic microorganisms but also often impart desirable sensory characteristics to the food. Yeasts have a long history of use in the production of different types of breads and fermented beverages including wine and beer. Some other examples of microfungi used in traditional foods include *Rhizopus* species to produce tempe and koji, *Aspergillus* species to produce miso and soy sauce, and *Penicillium* species to produce cheeses, such as Roquefort and Camembert (Table 2).

**Table 2:** List of Some Fungal Species Historically Used in Foods

phylum	genus	species	food product
mucoromycota	*Mucor*	*M. circinelloides*, *M. rouxii*, *M. indicus*	ragi, murcha, tempe
	*Rhizopus*	*R. microsporus*, *R. oligosphorus*, *R. oryzae*	tempeh, koji, nuruk, chu, murcha, tempe
ascomycota	*Neurospora*	*N. sitophila*, *N. intermedia*	oncom
	*Aspergillus*	*A. oryzae*	koji, miso, soy sauce, textured meat alternative
	*Fusarium*	*F. venenantum*	textured meat alternative (mycoprotein)
	*Penicillium*	*P. roquefortii*, *P. camembertii*	cheese
	*Tuber*	*T. magnatum*, *T. melanosporum*, *T. lyonii*	truffles
	*Morchella*	*M. esculenta*, *M. elata*, *M. rufobrunnea*	morel mushrooms
basidiomycota	*Agaricus*	*A. bisporus*	button mushrooms
	*Lentinula*	*L. edodes*	shiitake mushrooms
	*Boletus*	*B. edulis*	porcini mushrooms

The consumption of macrofungi, which include mushroom-forming fungi, also has a long history of human consumption and is still a major food source throughout the world. Mushrooms, including wild and cultivated mushrooms, are the fleshy reproductive structure of some macrofungi, most commonly from Basidiomycota including the common button and shitake mushrooms but also from Ascomycota, which includes morel mushrooms and truffles. In 2019–2020, alone, the U.S. mushroom crop totaled 816 million pounds, with a total sales value of $1.15 billion USD.^21^ Mushrooms are an abundant source of vitamins and other nutrients and are an important contributor to a healthy diet. Mushrooms are low in calories, fat, and sodium and are rich sources of beneficial food constituents such as fiber, selenium, potassium, riboflavin, niacin, and ergosterol that with ultraviolet (UV) light exposure produces vitamin D2.^22^

In addition to the well-known traditional fungi-based foods (i.e., mushrooms, some cheeses, and soy sauce), the use of fungal mycelium as a food source has been of increasing scientific and commercial interest, especially for certain species that have a good safety profile and can be utilized as a source of high-quality protein and desirable nutrient profile (Table 3). Moreover, mycelial species promoted for commercialization have fast growth rates, good texture, and flavor profile and can be produced in a sustainable and environmentally friendly manner. Thus far, commercially produced mycelium is most commonly derived from the cultivation of microfungi from Ascomycota; however, there are some examples of the utilization of mycelium from mushroom-forming macrofungi from Basidiomycota, such as *Lentinula edodes*, which produce shitake mushrooms. In this example, since the mycelium or vegetative growth form of the fungus is used in the food product, the product is not referred to as a mushroom, but rather a mycelium. The same is true for mold-forming microfungi used in food products. A mold is a reproductive structure of some microfungi, analogous to a mushroom being the reproductive structure of some macrofungi. If the mycelium of a microfungus is used in the food product, the product itself is not a mold, but rather mycelium from a mold-forming fungus. In contrast, a mold may form on some cheeses, so the term mold is accurate in those cases. Further complicating the nomenclature, the term mold often has negative connotations since most consumers associate the term mold with mycotoxins and mold allergies, even though edible mold-forming fungi are used to produce many commonly consumed foods, such as tempeh, miso, soy sauce, and some cheeses. Thus, there will be a need for consumer education on the differences between toxic molds, edible molds, and mycelium from mold-forming fungi. Moreover, as new mycofoods emerge, the documentation of their safety^23,24^ will further aid in consumer acceptance of these foods into the mainstream global food market.

**Table 3:** Commercial Examples of Protein-Rich Mycofoods Marketed as Meat Alternatives^83^

species	business founding	business location	company name	brand name	application
*Fusarium venenatum*	1985	United Kingdom	Marlow Foods Ltd.^84^	Quorn	textured meat alternatives
*Fusarium* strain flavolapis	2009	United States	Nature’s Fynd (Formerly Sustainable Bioproducts, Inc.)^84^	Fy Protein	textured meat alternatives cream cheese alternatives
				Fy	
*Neurospora crassa*	2014	United States	Emergy Inc. (Formerly Emergy LLC)^84^	Meati	whole-cut meat alternatives
				EatMeati	
*Aspergillus oryzae*	2017	United States	Prime Roots (formerly known as Terramino Foods)^84^	Koji	textured meat alternatives
*Lentinula edodes*	2017	Vietnam	Emmay^84^	Smiley Mushroom	whole-cut and textured meat alternatives

## Mycelium Flavor

Innovative approaches in food chemistry, including textural modifications and in-process flavor development, present an opportunity to design novel mycelium-based food products with enhanced flavor and texture. These technologies can help meet the consumer’s “flavor” and “texture” expectations of mycelium-based food products in a sustainable and environmentally friendly manner. Mycelium-based food products typically have a bland or slightly mushroom-like flavor profile, and as a result, most commercial products have added ingredients such as spices, yeast extract, or natural flavors added (i.e., chicken, beef, etc.). Accordingly, there is an opportunity for more consumer research to determine optimized flavor profiles that are the most appealing to consumers and develop technologies to enhance the flavor without the addition of natural flavors. One current area of research is the development of desirable in-process flavor, utilizing the knowledge of the fundamental biochemistry and flavor chemistry naturally present in different fungi. For example, different species of mushrooms can produce a wide variety of flavors such as *Boletus pallidoroseus*, with the aroma of beef bouillon, *Laetiporus sulphureus*, with the flavor and texture of chicken, *Boletus sensibilis* that smells like curry, and*Lactarius camphoratus* with a maple syrup aroma. Raw mushrooms contain a pool of aroma precursors (i.e., amino acids, peptides, and sugars) that when cooked react to generate odorants that elicit the unique flavor of the cooked mushroom. For example, the lobster mushroom, which is a *Russula* or *Lactarius* species of mushroom that has been parasitized by*Hypomyces lactifluorum*, has a prominent seafood-like flavor that develops after only after thermal treatment. The seafood-like flavor is hypothesized to be derived from odorless precursors present in the raw mushroom that upon heating generate seafood-like flavors. Slight differences in the amino acid composition can drastically affect the final flavor chemistry of the cooked product. Many fungi contain the flavor chemistry potential to generate a wide variety of flavors both endogenously and through thermal treatment. This knowledge may also be applied to mycelium-based food products; however, further research is needed in this area.

## Mycelium Composition

The Dietary Guidelines for Americans 2020–2025 recommends that an individual’s diet contain a variety of protein foods from both animal and nonanimal sources.^6^ Foods within the latter are complex whole foods, considerably lower in saturated fat and sodium, while providing dietary fiber, vitamins and minerals, and additional nonessential bioactives. While increased intakes of plant-based foods lower the risk for the development of chronic disease,^25^ protein quality can be a concern as the cell wall structure and the presence of antinutritional factors can limit both micronutrient and amino acid availability.^26,27^ The intake of a variety of nonanimal source proteins that ensure completeness of overall essential amino acid intake may overcome these issues. In addition, the provision of protein isolates can improve amino acid availability, but may lower the content of fiber and other beneficial nutrients and bioactives in comparison to the whole food source.^26,28^

Within the nonanimal protein realm, mycelium research to date shows promise for this food’s incorporation into a healthy diet. Similar to plant proteins, mycelium is low in total fat, which is primarily unsaturated, and a source of fiber (Table 4). On a dry matter basis, the protein content of fungi such as mycelium is on the order of 20–30%.^29^ Moreover, commercialized species such as *Fusarium venenatum* and *Neurospora crassa* are considered high quality in protein with a company-reported protein digestibility-corrected amino acid score (PDCAAS) at or near 1.0.^23,30^ This indicates that 100 g of protein from these products provide at or near 100% of the essential amino acids^31^ (Table 5). Moreover, the filamentous nature of mycelium allows for food production via fermentation into products that mimic the texture of meat.^32^ Mycelial protein is incorporated into a multilayered cellular wall structure of polysaccharides, predominately consisting of β-glucan and a smaller proportion of chitin in the innermost layer near the plasma membrane.^33^ Chitin, a homopolymer of β-1,4-linked N-acetyl glucosamine units, is the main fibrous polysaccharide found in insect cytoskeletons, fish scales, and fungi.^34^ The soluble fiber β-glucan from cereals comprise β-1,4 and β-1,3 linked glucose units, with the cholesterol-lowering effects of cereal β-glucan intake being well documented.^35^ For mycelium, the innermost cell wall predominately consists of either lineal β-1,3 glucan units or β-1,3 glucan units with β-1,6 linkages at branching points.^33^

**Table 4:** Raw Ingredient Comparison of Whole Mycelium, Whole Plants, and Animal Foods per 100 g^a^

		FAO/WHO 2013 PDCAAS scoring	mycofoods			plants		animals	
			mycoprotein^30^	mycelium, whole^39^*Neurospora crassa*)	portabella mushroom, raw^85^	chickpea, boiled (canned and rinsed)^86^	soybean raw^87^	beef, raw (filet)^88^	chicken, raw, breast meat only^89^
amount	g		100	100	100	100	100	100	100
water	g		NR	NR	92.8	66.9	67.5	72.5	73.9
calories	kcal		86	95	22	138	147	125	120
total fat	g		2.9	1.0	0.4	6.0	6.8	3.7	2.6
sat fat	g		0.6	0	0	0.2	0.8	1.3	0.6
mono Fat	g		0.5	NR	0.04	0.49	1.28	1.76	0.69
poly Fat	g		1.8	NR	0.30	0.96	3.2	0.15	0.42
sodium	mg		5.0	6.0	9.0	212	15.0	57.0	45.0
carbohydrates	g		3	7.4	3.9	22.9	11.0	0	0
fiber	g		6	5.3	1.3	6.3	4.2	0	0
sugar	g		0.5	0	0	0	0	0	0
protein	g		11	12.6	2.1	7.0	13.0	22.9	22.5
EAA									
histidine	mg/g protein	20	35.5	25.8	27.488	27.7	26.8	43.1	37.3
isoleucine	mg/g protein	32	51.8	43.2	38.9	43.0	43.8	52.8	48.9
leucine	mg/g protein	66	86.4	74.9	61.6	71.4	71.2	98.3	82.7
lysine	mg/g protein	57	82.7	79.5	57.8	67.2	59.6	110.0	96.0
methionine + cysteine	mg/g protein	27	NR	32.5	18.5	26.8	21.2	41.7	36.5
phenylalanine + tyrosine	mg/g protein	52	NR	70.7	42.7	78.8	80.8	87.6	76.4
threonine	mg/g protein	31	55.5	49.4	47.9	37.4	39.7	53.3	44.9
tryptophan	mg/g protein	8.5	16.4	15.1	16.6	9.8	12.1	12.4	12.6
valine	mg/g protein	43	54.5	63.1	36.0	42.3	44.3	55.5	51.6
PDCAAS			0.99	1.0	N/A	0.5^90^	0.85^91^	1.0	1.0
calcium	mg		48	15.0	3.0	43.0	197.0	5.0	5.0
iron	mg		0.39	0.9	0.3	1.0	3.5	2.2	0.4
magnesium	mg		NR	23.0	NR	24.0	65.0	26.0	28.0
phosphorus	mg		290	340.0	108.0	80.0	194.0	233.0	213.0
potassium	mg		71	315.0	364.0	109.0	620.0	389.0	334.0
zinc	mg		7.6	4.5	0.5	0.6	1.0	6.1	0.7
thiamin	mg		0.1	0.1	0.1	0	0.4	0.1	0.1
riboflavin	mg		0.3	0.9	0.1	0	0.2	0.2	0.2
niacin	mg		NR	6.7	4.5	0.1	1.7	5.7	9.6
pantothenic acid	mg		NR	3.2	1.1	NR	0.1	NR	1.5
folate	μg		114	150.0	28.0	41.0	165.0	4.0	9.0
choline	mg		180	80.0	21.2	NR	NR	NR	82.1
vitamin B12	μg		0.71	NR	0.1	0	0	2.7	0.2

a EAA, essential amino acids; mono fat, monounsaturated fat; NR, not reported; poly fat, polyunsaturated fat; PDCAAS, protein digestibility-corrected amino acid score; sat fat, saturated fat.

**Table 5:** Self-Reported Mycelium Protein Digestibility-Corrected Amino Acid Score (PDCAAS)^a^

**company**	**brand name**	**mycelium species**	**reported PDCAAS**
EatMeati	Mushroom Root Protein	*Neurospora crassa*(84)	1.00
MYCO Technology	FermentIQ Protein	pea and rice protein fermented with shiitake mycelium^92^	1.00
Quorn	Mycoprotein	*Fusarium venenatum*(92)	0.99
Eternal Mycofoods	N/A	*Fusarium venenatum*(92)	0.92
Nature’s Fynd	Fermented Microbial Protein or Fy Protein	*Fusarium* strain flavolapis	0.92
		*Fusarium novum. yellowstonensis*(84)	

a N/A = not applicable.

Depending on the growth substrate, the micronutrient profiles of mycelium can vary (Table 4), yet may be a dietary vehicle for the delivery of a number of essential micronutrients of concern, particularly for population groups that solely consume a plant-based diet, and includes iron, zinc, and vitamin B12.^36,37^ Indeed, a serving of certain commercially available mycelial products can be considered a high source of zinc, folate, copper, riboflavin, niacin, and pantothenic acid, providing at least 20% of the daily value, while a good source of iron (Table 4).^30,38,39^ Additionally, mycelium is low in phytate, which can make it a more bioavailable nonanimal protein source of micronutrients such as zinc.^40^ Although promising, data from dietary intervention trials are needed to confirm the bioaccessibility of essential micronutrients from mycelium.

Depending on the species and growing conditions, mycelium can be a source of a number of bioactive compounds. This includes ergothioneine, a derivative of histidine and betaine that exists as a tautomer of thiol and thione. At physiological pH, thione is dominant, making ergothioneine less reactive and resistant to autoxidation.^41^ Ergothioneine can be found in a variety of foods, most likely derived from the presence of fungi either near or at the root level.^42^ While the biological role of ergothioneine is still being defined, low ergothioneine levels have been associated with age-related chronic and neurodegenerative diseases.^41^ Both macro- and micro fungi produce additional bioactives and pigments as a protective response against UV-light-induced oxidative stress. This includes carotenoids, such as neurosporaxanthin and γ-carotene, produced from Neurospora^43,44^ and ergosterol or vitamin D2.^29^

On a global basis, reducing food waste and loss is of interest for long-term environmental sustainability as well as food and health security. Upcycling of food waste streams provides for the reincorporation of nutrients into the food system, and for an industry sector, creates a resilient circular bioeconomy.^45^ Examples include the mycelial fermentation of soybean cake and tofu waste, into oncom and tempeh, resulting in increased protein content and nutrient bioaccessibilty.^46^ For livestock, mycelial fermentation of agricultural waste streams reintroduces fiber and protein back into the food system.^47^ Moreover, depending on the waste stream, mycelial fermentation allows for the incorporation of bioactive peptides and plant-derived secondary metabolites, such as flavonoids, known for their health-promoting properties into mycofoods.^48,49^

## Mycelium and Health

A number of studies have reported positive impacts of mycelial extracts on the immune system, cancer, and cirrhosis in *in vitro* and animal models and human participants.^29,47,50,51^ Focusing specifically on mycelial intake as a whole food, a limited number of dietary intervention trials suggest positive impacts on glycemic response.^52^ In an oral glucose tolerance test, healthy participants consumed either a beverage providing 17 g of mycoprotein and 75 g of carbohydrate (50 g as glucose) or an energy, protein, and carbohydrate-matched control beverage. From baseline to 60 min post beverage intake, the participants had an 8.75 and 20% reduction in their area under the curve (AUC) glucose and insulin responses, respectively.^53^ No significant intervention effects for postprandial glucose were observed when healthy men consumed beverages providing 20 g of milk or mycoprotein (0.7 and 4.0 g total carbohydrate, respectively). However, plasma hyperinsulinemia was slower and more sustained compared to a similar amount of milk protein.^54^ Similar to this, postprandial glucose response was not significantly impacted in individuals with an overweight or obese BMI who were provided several different levels of mycoprotein (at 44, 88, and 132 g) or a protein-matched and isoenergetic amount of chicken in risotto (delivering 25–30 g of total carbohydrate).^55^ In the same trial, insulin sensitivity as measured by the Matsuda index was significantly greater at the highest level of mycoprotein intake compared to the same amount of chicken, while the insulinogenic index, a measure of beta-cell output, was 18, 15, and 30% lower with low, medium, and high intakes of mycoprotein, respectively, compared to chicken.^55^

Beyond metabolic responses, mycelium intake suppresses appetite and energy intake,^52^ with a noted need for data over prolonged periods of intake.^52^ In this regard, early dietary intervention trials enrolling individuals with slightly elevated cholesterol levels demonstrate the potential of mycelium intake to lower cholesterol levels. In a 3-week metabolic study, diets providing 190 g of mycelium (*Fusarium venenatum*) per day significantly lowered LDL cholesterol by a mean difference of 21% for those consuming a diet matched for calories with the provision of animal protein.^56^ Similar results were observed in a follow-up trial of free living adults, who consumed cookies with or without approximately 130 g of mycelium equivalents for 8 weeks.^57^ Taken together, these data suggest the potential of mycelium to have positive impacts on cardiometabolic health; however, these studies are limited to one mycelial species and will need confirmation as products are developed from additional species.^58^ Moreover, for the most part, studies to date have been under controlled dietary conditions, with more data needed on the potential health impacts of currently available commercial products when they are incorporated into the daily diet.

The abovementioned lipid-lowering effects may be due to increased intakes of the mycelium-derived fiber. Improvements in gut health and lower LDL cholesterol levels have been reported with chitin supplementation. Toward this end, there is considerable interest in fungi-derived chitin-glucan complexes.^34,59^ Animal models to date suggest that fungal-derived β-glucan may produce similar results as their cereal-derived counterparts.^60^ Fermentable fibers such as β-glucan can produce short-chain fatty acids that suppress 3-hydroxy-3-methyl-glutaryl-CoA reductase (HMGR), the rate-limiting enzyme for cholesterol synthesis, along with activating sterol regulatory element-binding proteins (SREBP)-2 to increase hepatic LDL-receptor gene expression that helps clear cholesterol.^45^ Both mycelium and mycelium-derived fiber increased propionate and butyrate production in an in vitro fermentation model.^61^ It is important to note that the glycosidic bonds can differ between plant fungi kingdoms, and food processing may influence any functional effect. Indeed, recent *in vitro* data suggest that although β-glucans are released to a greater extent from plants, β-glucan release from mycelium can be enhanced with cooking.^62^

The anabolic response, or muscle protein synthesis, is stimulated with the rise of plasma essential amino acids, particularly leucine, while muscle protein breakdown (catabolism) is inhibited by hyperinsulinemia in the postprandial period.^54^ Data collected to date suggest that amino acid bioavailability, and ultimately the stimulation of muscle protein synthesis, is considerably different with mycelium intake compared to that of animal-based protein. First, animal proteins such as milk produce a rapid (within 30 min) rise in essential amino acids coupled with hyperinsulinemia. In contrast, 18 g of protein from *Fusarium venenatum* produces a similar but more sustained hyperinsulinemia and hyperaminoacidemia compared to 16 g of milk protein intake.^54^ These observations are potentially functionally significant, as resting and post exercise muscle protein synthesis rates were indeed greater with mycelium protein intake compared to milk protein intake.^63^

## Mycelium and Environmental Impact

The UNSDG2 aims to end hunger, and achieve food and nutrition security within a sustainable agricultural system by 2030.^64^ Achievement of this goal was already off track prior to the COVID-19 pandemic that escalated already deteriorating food security, with 12% of the global population estimated as severely food insecure in 2020.^64^ The multidisciplinary approach of UNSDG2 to address worldwide hunger includes the development of agricultural systems that are sustainable and regenerative, in that they maintain ecosystems and protects resources as opposed to industrial agricultural systems that lead to environmental degradation, including air and water pollution, soil depletion, and diminishing of biodiversity.^65^ For protein, forecasts are for a 50% increase in meat production on twice as less arable land by 2050 in order to supply global nutritional demand.^66^ To meet both growing demand and increasingly severe constraints, food companies must drive sustainable innovation to produce large amounts of high-quality, safe protein that preserves limited resources, including land and water. Moreover, the developed products will need to be accessible and affordable to the global population, with the goal of decoupling the cost and ability to eat a healthy diet with persistent high levels of income inequality.^64^

The term novel and future foods (NFFs) describes a group of foods that utilize nontraditional agricultural practices to produce a source of protein that addresses the environmental impacts of food. Ingredients grouped under this category include but are not limited to mycelium, insect meal, microalgae, and cell-cultured meat.^67^ In a recent environmental impact model that also optimized for nutritional adequacy and feasibility of intake, NNFs yielded substantial reductions in environmental pressures related to land use (LU), water use (WU), and global warming potential (GW) when compared to traditional European dietary patterns.^68^ Environmental pressures against the traditional European diets, when including insect meal, cultured meat and milk, algae protein, mycelium, and bacteria as NFFs, had a predicted mitigation of 87% LU, 84% WU, and 83% GW. Although an optimized vegan diet (VEG) had the largest impact on GW (85% mitigation), NFFs had a greater impact on WU and LU compared to VEG (83% and 81%, respectively).^69^ However, this study represents the eating habits of 8% of the worldwide population, demonstrating the need for research that includes the impact of NFFs within other dietary patterns, societies, and ethnicities.^68^

When one aims to reduce the environmental impact of a diet, nutrition quality can be a concern, particularly when one food group for another. Shifting diets away from animal-sourced foods (ASFs) to a more plant-based diet may provide less stress to environmental resources. However, plant foods can be less favorable in their essential nutrient composition and bioaccessibility compared to ASFs.^70,71^ One option to overcome this limitation is the incorporation of NFFs into a plant-based or a limited ASF dietary pattern that still promotes a reduction in environmental stress.^69^ In the aforementioned study by Mazac et al., replacing ASFs with NFFs in a traditional European dietary pattern not only reduced environmental impacts but also met nutrient needs.^69^ This study also indicates that the inclusion of smaller amounts of ASFs by optimizing the current diet to recommended levels of intake will also lower the environmental impact, and minimal inclusion of NFFs will help meet nutrition needs. For the NSFs utilized in this study, mainly insect meal, cultured milk, microbial protein, and mycelium demonstrated the best nutrition content and environmental impact; however, lifecycle analysis assessments on NFFs are limited at this time and further research is recommended.

As described above, mycelium is a good quality protein, providing essential micronutrients similar to those of meat. Current modeling also suggests that replacing ASFs with mycelium can have a positive impact on the environment.^72,73^ Accounting for all outputs of food production—feed production, manure storage/spreading, enteric methane, and processing and packaging of the finished product—carbon footprint estimates of mycofoods were 10 and 4 times less compared to beef or chicken, respectively.^72^ For WU per gram of protein produced, mycelium was 10 and 3 times less than beef and chicken, respectively, along with 10 and 2 times less LU than beef and chicken, respectively.^72^ More recently, Humpenöder et al. estimated that the per capita substitution of 20% of ruminant-derived protein for mycoprotein offsets future LU and CO2 emissions by half by 2050, while also lowering methane emissions.^73^ While promising, studies are limited to one species of mycelium and will need to account for variations in the technologies utilized to grow mycelial protein and the ingredients used in their production.

Data to date suggest that the incorporation of mycelium into a dietary pattern can lend toward reducing the negative impacts of the food system on the environment. However, world hunger and food insecurity are inextricably linked between social inequality and access to healthy food options; therefore, the key to replacing ASFs within the food system will be the availability and affordability of any alternative protein source. While healthy and sustainable diets, such as the EAT-Lancet have been proposed, the cost of such a diet is predominately driven by plant-based foods, with a large percentage of per capita household income (up to 89%) needed to afford this dietary pattern in lower versus higher income countries.^74^ Protein affordability is dependent on costs of production; ASF can take several weeks to years, while the production of plant-sourced proteins through traditional agricultural practices can take several months with the potential for weather-related loss in crop production (Table 6). Given the nutritional value that is comparable to that of ASF, but with reduced environmental impact, NFFs such as mycelium are an appealing option. As innovations in this field work toward the production of these products at scale and at a lower cost, mycelium is appealing as a nutrient dense source of protein providing fiber and essential micronutrients that can be grown in a relatively short period. Indeed mycelium research indicates that protein production can happen in days instead of months or years; however, the strain, media, and growing conditions all play a role in predicting the growth rate.^75^

**Table 6:** Production Cycle for the Source of Protein

protein	time	influence production cycle	source
beef cattle, average age at slaughter	1.8 years average (up to 3 years)	feed, breed, pastures, vaccinations/dz management	(93,94)
pork	6 months	feed, breed	(95)
chicken	7 weeks to 3–5 mo	feed, breed	(96)
rice protein	120 days	consistent irrigation, soil health, planting timing, variety	(97)
pea protein (yellow peas)	80–90 days	rain, irrigation, temperature, soil health, sunlight	(98)
soybean (relative maturity is dependent on time of planting, phenotype, and seasonal influences)	45–65 days	rainfall, climate	(99)
chickpea	100 days average (83–125)	rainfall, climate, variety dependent	(100)
almonds	3 years for first crop from planting and harvest once a year	rainfall, temperature, variety	(101)
peanut (*Arachis hypogaea* L.; Virgínia group)	140–150 days	planting timing, day length, temp, rain, wind, variety	(102)
mycelium	2–6 days	species, growing method	

For NFFS such as mycelium to be part of the solution to solve hunger and food insecurity, there is a need for an investment in resources and infrastructure to scale production. These resources include finding ways to reduce production costs to make these ingredients affordable to all, in addition to interventions that educate and promote the use of these ingredients as a staple in the diet.^61^ A recent survey of European consumers reports that 56% of respondents had not heard of the term “fungal or mycoprotein”.^76^

## Mycelium and Food Technology To Grow Mycofoods

Cultivating mycelium offers a variety of different technologies and methods, some having been used for hundreds of years and others having been developed in the past century. The two most common mycelium production systems are *solid-state* and *submerged* fermentation.^11^ In solid-state fermentation, mycelium is grown on a solid substrate that is usually a food source such as a grain or a legume, with the mycelium-permeated substrate harvested and used as is or processed further. This method has been commonly employed to make products such as tempeh.^77^ In submerged fermentation, mycelium is grown primarily in liquid media with specific nutrients, and often separated by filtration or by other means prior to use or can undergoes further processing.^11^ Large-scale submerged fermentation has been practiced with mycelium starting with the manufacturing of penicillin and has progressed with developments in fermentation for more advanced products such as food additives and enzymes.^78^ Some practices of mycelium submerged fermentation are being used for direct human food cultivation.^79^

Solid-state, submerged, or other hybrid methods each have their own advantages. For instance, while solid-state systems are generally thought to have lower upfront capital investments, the volumetric productivities and speed of growth in submerged fermentation makes this to be the mycelium cultivation technology of choice. In order for mycelium to become a sustainable human nutrition solution and alleviate global hunger, there is a need for substantial investment in both solid-state and submerged fermentation to unlock significantly more mycelium production capacity. With this said, a recent techno-economic analysis suggests that mycoprotein production can be on par with globally relevant sources, such as beef, utilizing existing manufacturing technologies.^80^ Therefore, further advances may enable mycoprotein production to surpass beef and approach even cheaper animal-based commodity proteins such as poultry. Thus, the opportunity to improve global health outcomes through nutrition that achieves key measures of environmental sustainability via increased production of mycoprotein seems to be based on a firm foundation regarding economics and positive returns on related investments.

Technology advances have enabled the production of mycelium into scalable biomass for use as an alternative sustainable food product. With its quality protein, essential micronutrient profile, and lower impacts on land and water, plus reduced greenhouse gas production, incorporation of mycofoods into food systems can aid in the achievement of UNSDG2 goals to end hunger and achieve food and nutrition security within a sustainable and regenerative agricultural system. Although promising, the limited data on the potential health impacts of mycelium intake need to include confirmatory data across mycelial species. This includes data from diverse population groups across the lifespan. In this regard, while initial studies on the anabolic effects of mycelium are promising, there is also a need for data on the ability of mycelium protein intake in support of human growth. Future considerations also include adapting production of mycofoods to utilize local resources and create education programs that demonstrate how these ingredients can fit with current cultural practices and meet consumer taste preferences. The ultraprocessed nature of many current plant-based meat mimetics including the addition of sodium, sugar, saturated fat, and additives to enhance flavor, texture, and color is a concern for both health professionals and consumers.^81,82^ The filamentous nature and nutrient density of certain types of mycelium, coupled with the potential for innovations in fungi flavor, can enable mimetic product development that requires fewer additives for flavor and texture, with less sodium and low saturated fat. Moreover, mycelium’s unique properties enable its use as an ingredient in other product formulations and represent an opportunity to reduce the need for other additives within alternative plant protein-based recipes. Therefore, mycelium represents a significant opportunity to help usher in a new era of product development produced at scale that is considered healthy yet with fewer ingredients and has a sensory profile that is complex with depth. Once achieved, mycelium will certainly be appealing as an environmentally friendly, nutrient dense protein source that can aid in the reduction of global hunger.
